# Joint effects of voluntary participation and group selection on the evolution of altruistic punishment

**DOI:** 10.1371/journal.pone.0268019

**Published:** 2022-05-04

**Authors:** Hoon C. Shin, Sechindra Vallury, Marco A. Janssen, David J. Yu

**Affiliations:** 1 Lee Kuan Yew School of Public Policy, National University of Singapore, Singapore, Singapore; 2 Center for Behavior, Institutions, and the Environment, Arizona State University, Tempe, Arizona, United States of America; 3 WA Franke College of Forestry & Conservation, University of Montana, Missoula, Montana, United States of America; 4 School of Sustainability, Arizona State University, Tempe, Arizona, United States of America; 5 School of Complex Adaptive Systems, Arizona State University, Tempe, Arizona, United States of America; 6 Lyles School of Civil Engineering, Purdue University, W. Lafayette, Indiana, United States of America; 7 Department of Political Science, Purdue University, W. Lafayette, Indiana, United States of America; University of Electronic Science and Technology of China, CHINA

## Abstract

It is puzzling how altruistic punishment of defectors can evolve in large groups of nonrelatives, since punishers should voluntarily bear individual costs of punishing to benefit those who do not pay the costs. Although two distinct mechanisms have been proposed to explain the puzzle, namely voluntary participation and group-level competition and selection, insights into their joint effects have been less clear. Here we investigated what could be combined effects of these two mechanisms on the evolution of altruistic punishment and how these effects can vary with nonparticipants’ individual payoff and group size. We modelled altruistic punishers as those who contribute to a public good and impose a fine on each defector, i.e., they are neither pure punishers nor excluders. Our simulation results show that voluntary participation has negative effects on the evolution of cooperation in small groups regardless of nonparticipants’ payoffs, while in large groups it has positive effects within only a limited range of nonparticipants’ payoff. We discuss that such asymmetric effects could be explained by evolutionary forces emerging from voluntary participation. Lastly, we suggest that insights from social science disciplines studying the exit option could enrich voluntary participation models.

## Introduction

Human cooperation with non-kin in large groups is an evolutionary puzzle because cooperators voluntarily bear individual costs of cooperating to benefit non-relatives who do not pay the costs [1–3]. Altruistic punishment may explain the question of how costly cooperation persists. Laboratory [4, 5] and ethnographic [6, 7] evidence shows that people are willing to punish defectors at a cost to themselves without any material gain even in anonymous, one-shot interactions and that such punishment contributes to sustaining human cooperation. However, another issue must be addressed to accept the argument that altruistic punishment explains the evolution of cooperation: how can altruistic punishment evolve despite the payoff disadvantage of punishers? From the evolutionary viewpoint, this is also puzzling because natural selection works against punishers who incur individual costs of punishing defectors and favors those who do not pay the costs [2]. Note that altruistic punishers of our interest are those who impose a fine on each defector as well as contribute to a public good. In this sense, the type of altruistic punishment we explore is neither pure punishment (i.e., not contributing to a public good but punishing defectors) [8, 9] nor social exclusion (i.e., preventing defectors sharing collective benefit from a public good) [10–12].

Previous studies have shown two distinct paths toward the evolution of altruistic punishment: group selection and voluntary participation. A fundamental logic of group selection is that fitness variations owing from group-level properties cause differential reproductive success [13, 14]. A corollary is that more cooperative groups are more likely to survive selection pressures such as intergroup competition (e.g., warfare) and success or failure in sustaining natural resources [15]. The theoretical model by Boyd et al. [1] builds on this notion to create an artificial society of multiple groups, each of which produces a local public good beneficial for its own members only. The model then shows that group selection helps altruistic punishment to spread across the society despite the initial dominance of defection in most groups, thereby demonstrating its plausibility for explaining the evolution of altruistic punishment in large groups. While group selection is certainly a key to probing the origin of altruistic punishment, this group selection model has been tested in the absence of a general feature found in most real-world social interactions—voluntary (or optional) participation. In many situations, individuals can freely decide whether or not to participate in a joint endeavor. Boyd et al.’s model assumes that individuals of each group have no option to withdraw from public goods provision.

Such a compulsory participation assumption underlying the group selection model has been often eased in behavioral experiments [16–18] and evolutionary game theories [19–30]. In these studies, individual agents may opt out of prisoners’ dilemma or public goods games to receive a constant payoff unrelated to the others’ strategies. Surprisingly, allowing this simple option led to persistence of altruistic punishment in evolutionary models [2, 31]. The finding is that, compared to obligatory participation, voluntary participation is more effective in helping altruistic punishment to emerge from within a large group. Examples of the voluntary participation could be collecting mushrooms instead of joining a collective hunt in a tribal society [31] or using a private tube-well instead of a collectively-managed canal [32]. This insight about voluntary participation, however, remains tested only for a single group environment void of group-level fitness variation and selection pressures [33]. What are joint effects of voluntary participation and group selection on the evolution of altruistic punishment? Generalizable insights into this question have been elusive and identified as a gap by a bibliographic analysis of the mechanisms for the evolution of cooperation [33].

Addressing this question is the aim of the current paper. We do so by analyzing a multilevel selection model of public goods provision in which group selection and voluntary participation co-occur. In our model, individuals interact to select more lucrative strategies among 1) defection, 2) contributing to a local public good without punishing defectors (non-punishing cooperation) and 3) contributing to both a local public good and punishment (punishing cooperation), and 4) nonparticipation. In line with the existing studies of voluntary participation [2, 3, 31], we define nonparticipation as neither producing a local public good nor consuming the public good produced by others. Along with the individual-level payoff-biased selection, our model also allows more cooperative groups to be culturally selected through group-level interaction.

Given the multilevel selection above, it follows that the evolution of altruistic punishment in our model should be influenced by a complex, dynamic, and long-term interaction of at least three evolutionary forces described next. First, a *defector-decreasing* force could come into play if defectors choose to opt out of public goods provision. This force will help altruistic punishment spread within a group because the cost of punishing, which is proportional to the number of defectors, decreases. Second, a *punisher-decreasing* force could also emerge if punishers choose to withdraw from the public good project. This force will increase defectors because the cost of being punished, which is proportional to the number of punishers, decreases. The rise in defectors, which increases the cost of punishing, will likely further reduce punishers. Third, *group-selection* force works between groups to affect group-level properties associated with group-beneficial outcomes, which is the spread of punishers. Although these forces can all potentially shape the outcome of multi-level selection, it remains unclear under what conditions their combined effects can enhance levels of cooperation (including non-punishing and punishing cooperation) in the long run.

We speculate that nonparticipants’ payoff is a critical parameter shaping the combined effects on the evolution of cooperation under the presence of the evolutionary forces above. If the payoff is too low, both defectors and punishers will not become nonparticipants. This means that both *defector-* and *punisher-decreasing* forces are so ineffective that the outcome of voluntary participation can be similar to the outcome of compulsory participation. By contrast, if nonparticipants’ payoff is too high, both defectors and punishers will make a switch to nonparticipants. This will lead both the forces to become so active that nonparticipants can outnumber participants in public goods provision. A rapid, massive reduction in participants due to the high payoff to nonparticipation will likely make voluntary participation inferior to compulsory participation in terms of levels of cooperation. Lastly, if nonparticipants’ payoff is somewhere between the low and the high, nonparticipation might neither dominate nor become extinct in the long run. Hence, the *defector-decreasing* force could keep pace with the *punisher-decreasing* over time. In the midst of this interaction, *group-selection* force will also contribute to increasing punishers at the group level. This means that voluntary participation models have both individual- and group-level forces enabling the evolution of cooperation while compulsory participation models only have the group-level force that helps cooperative behaviors spread. Therefore, one can expect that voluntary participation could achieve a higher frequency of cooperation in the long run than compulsory participation. Putting these pieces together, we hypothesize that a curvilinear relationship exists between nonparticipants’ payoff and the long-run frequencies of cooperation in our multilevel selection model of public goods provision.

## The model

Our multilevel selection model builds on Boyd et al. [1]’s group selection model where individuals are not allowed to opt out of public goods provision. We added the option of nonparticipation to their model, thereby introducing multiple scenarios characterized by the availability of voluntary participation and levels of nonparticipants’ payoff. Such scenarios were simulated to test the joint effects of voluntary participation and group selection on levels of cooperation. In each scenario, there is a large population consisting of 128 groups of size *n*. Each group has three behavioral types of participants in the public good game: defectors who do not contribute to the public good but exploit the contributions of the other participants; contributors who contribute but do not punish the defectors; and punishers who not only contribute but also punish the defectors. The definitions of the three behavioral types are based on two previous studies [1, 2] that we based our multilevel selection model on. Note that an initial introduction of the option of nonparticipation to group members is attributed to mutation, i.e., participants in public goods provision flip to nonparticipants with probability *μ* (see Table 1). In this study, the term ‘cooperators’ represents a group of both contributors and punishers.

**Table 1:** Default parameter values.

Parameter	Description	Default value
*N*	Number of groups	128
*n*	Number of agents in each group	Varying (20 to 120)
*b*	Benefit if everyone cooperates	0.5
*c*	Cost of cooperation	0.2
*p*	Cost of being punished	0.8
*k*	Cost of punishing	0.2
*m*	Rate of mixing between groups (for individual imitation)	0.01
*μ*	Mutation rate	0.01
*s*	Rate of group pairing (for cultural group selection)	0.015
*e*	Erroneous defection rate	0.02
*Ω*	Nonparticipants’ payoff	Varying (0.80 to 1.30)

Note: All default values of the parameters (except for *n* and *Ω*) are based on Boyd et al. [1].

The payoff of a contributor is 1 + *bx* − *c*, where *x* is the fraction of cooperators in the group, and *c* is the cost of producing a public good. The term 1 + *bx* represents that a total benefit from the public good is proportional to the fraction of cooperators. The payoff of a defector is 1 + *bx* − *py*, where *y* is the fraction of punishers in the group and *p* is the cost of being punished. The term *py* indicates that the total cost of being punished increases as the fraction of punishers increases. This is because each defector is punished by every single punisher. These two payoff functions are common in both compulsory and voluntary participation scenarios. However, allowing voluntary participation makes a slight change in the payoff of a punisher. In a compulsory participation scenario, a punisher’s payoff is 1 + *bx* − *c* − *k*(1 − *x*), where *k* is the cost of punishing each defector. The term *k*(1 − *x*) implies that the total cost of punishing is proportional to the fraction of defectors because each punisher punishes every single defector. The option of nonparticipation changes the payoff function to 1 + *bx* − *c* − *k*(1 − *x* − *z*), where *z* is the fraction of nonparticipants. The term *k*(1 − *x* − *z*) signifies that the total cost of punishment is determined by the fraction of defectors who are neither cooperators nor nonparticipants.

While participants’ individual payoff varies with endogenous population dynamics, we assume that nonparticipants’ payoff is exogenously fixed. This assumption is in line with prior studies of voluntary participation [25, 31, 34]. The amount of nonparticipants’ payoff affects the relative fitness of participants in the public goods interaction. Our aim is to explore whether there is a certain range of nonparticipants’ payoff within which voluntary participation leads to a higher frequency of cooperation than compulsory participation. To do so, in each simulation we set nonparticipants’ payoff to be one of a contributor’s possible payoffs ranging from 1 − *c* (when *x* = 0) to 1 + *b* − *c* (when *x* = 1). Once a certain amount of nonparticipants’ payoff is given to a simulation, it is constant throughout the simulation.

Our compulsory and voluntary scenarios follow Boyd et al. [1]’s initial set-up and five sequential stages in each time period. Initially, one group has only punishers and the remaining 127 groups consist of all defectors. The following five events occur sequentially in each time period. First, cooperators in a group contribute to producing a local public good with probability 1 − *e* and defect with probability *e*. Defectors defect at all times without such an error. Second, punishers in a group reduce the payoff of each group member who defected during the first stage. After the second stage, group members imitate higher payoff individuals. More specifically, members in a group encounter another member from their own group with probability 1 − *m* and a member from another randomly chosen group with probability *m*. A member *i* who encounters a member *j* imitates *j* with probability *w*_*j*_/(*w*_*j*_ + *w*_*i*_), where *w*_*q*_ is the payoff of member *q* in the game, including the costs of punishing and being punished. This payoff-biased individual imitation leads to not only the spread of higher payoff behaviors within group, but also diffusion of the behaviors between groups with probability *m*. During the fourth stage, group selection occurs. Each group is randomly paired with one of the other groups with probability *s*. Their interaction results in one group taking over another group. The probability that group *i* takes over group *j* is 0.5{1 + (*x*_*i*_ − *x*_*j*_)}, where *x*_*g*_ is the frequency of cooperators in group *g*. This means that the group with more cooperators is more likely to take over another group with fewer cooperators. As a result, cooperation is the sole target of the resulting cultural group selection process. Finally, there is a small change of mutation for each member with probability *μ* (e.g., defectors flip to contributors, i.e., nonpunishing cooperators). To facilitate the replication of the model, we provide the ODD (Overview, Design concepts, Details) protocol [35] and the NetLogo code in (see S1 Text and S1 Code).

## Results

**Fig 1:**
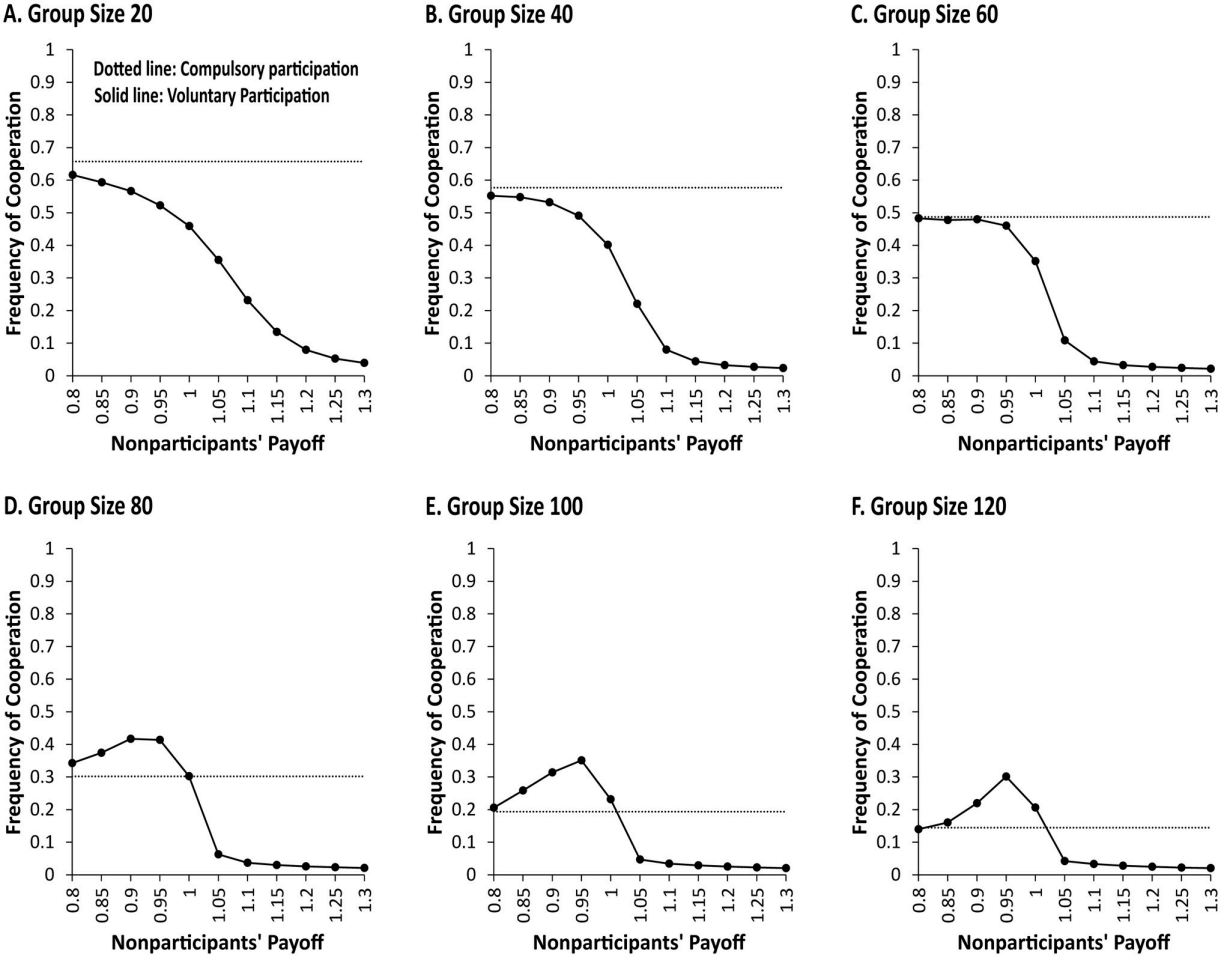
Asymmetric effects of voluntary participation on the long run average frequency of cooperation. Dotted lines show the simulation results of 100 runs of the compulsory participation scenario, and solid lines the results of the voluntary participation scenarios. Asymmetric effects are found in two dimensions, i.e., nonparticipants’ payoff and group size. First, only within a certain range of nonparticipants’ payoff (between 0.8 and 1.0), voluntary participation leads relatively large groups (*n* = 80, 100, *and* 120) to achieve higher frequencies of cooperation than compulsory participation. Note that the highest frequency of cooperation is observed when nonparticipants’ payoff (*Ω*) is 0.95 which is the same as a contributor’s payoff in a group where the faction of cooperators is 30% (*x* = 0.3); and the frequency of cooperation drops sharply when nonparticipants’ payoff increases to 1.05 which is the same as a contributor’s payoff in a group where the fraction of cooperators is 50% (*x* = 0.5). Second, such positive effects of voluntary participation are not observed in relatively small groups (*n* = 20, 40, *and* 60) regardless of changes in nonparticipants’ payoff. The data used in this figure are provided in S1 Data.

To compare the long run results of compulsory and voluntary participation scenarios, each one was simulated using the same set of parameter values that Boyd et al. [1] chose to capture cultural evolution (see Table 1). We also followed Boyd et al. [1] to identify a span of simulation time (time period = 1 year) and a way of calculating the long run average frequency of cooperation. For a range of group sizes (*n*), each scenario was run for 2,000 time periods. Considering an initialization period, we report the long run average frequency of cooperation over the last 1,000 time periods of 100 simulations while Boyd et al. [1] ran 10 simulations. The long run average results are plotted in Fig 1.

Fig 1 shows two asymmetric effects of voluntary participation on the evolution of cooperation. One of the asymmetric effects is identified in terms of nonparticipants’ payoff and the other in terms of group size. First, as shown in Fig 1D–1F, there is a certain range of nonparticipants’ payoff (between 0.8 and 1.0) within which voluntary participation leads to higher frequencies of cooperation than compulsory participation for relatively large groups (*n* = 80, 100, *and* 120). As participants’ payoff increases beyond the range, however, the long run average frequency of cooperation drops sharply and becomes much lower than the frequency of cooperation emerging from compulsory participation. This is an asymmetric effect observed in the dimension of nonparticipants’ payoff, supporting our hypothesized, culvilinear (or inverted U-shaped) relationship between nonparticipants’ payoff and levels of cooperation.

Second, such a payoff-based asymmetric effect is observed only in relatively large groups while not in relatively small groups (*n* = 20, 40, *and* 60). From the viewpoint of group size, this is another asymmetric effect of voluntary participation on the evolution of cooperation. Fig 1A–1C do not show the concave relationship between nonparticipants’ payoff and the long run average frequency of cooperation. An increase in nonparticipants’ payoff is likely to lead the frequency of cooperation to become lower than the frequency of cooperation observed in the compulsory participation scenario. This means that, for relatively small groups, voluntary participation tends to decrease the frequency of cooperation regardless of nonparticipants’ payoff levels.

Depending on the individual imitation of higher payoff behaviors, Hauert et al. [31] showed that “if individuals have the option to stand aside and abstain from the joint endeavor, this paves the way for the emergence and establishment of cooperative behavior” [31]. Their finding suggests the possibility that the prosocial norm within a group can emerge from payoff biased imitation at the individual level. We took their voluntary participation model a further step by modeling cultural group selection that could contribute to the evolution of cooperation. Our simulation results show that combined with group selection, voluntary participation can either facilitate or suppress the establishment of cooperative behaviors when nonparticipants’ payoff and group size vary.

**Fig 2:**
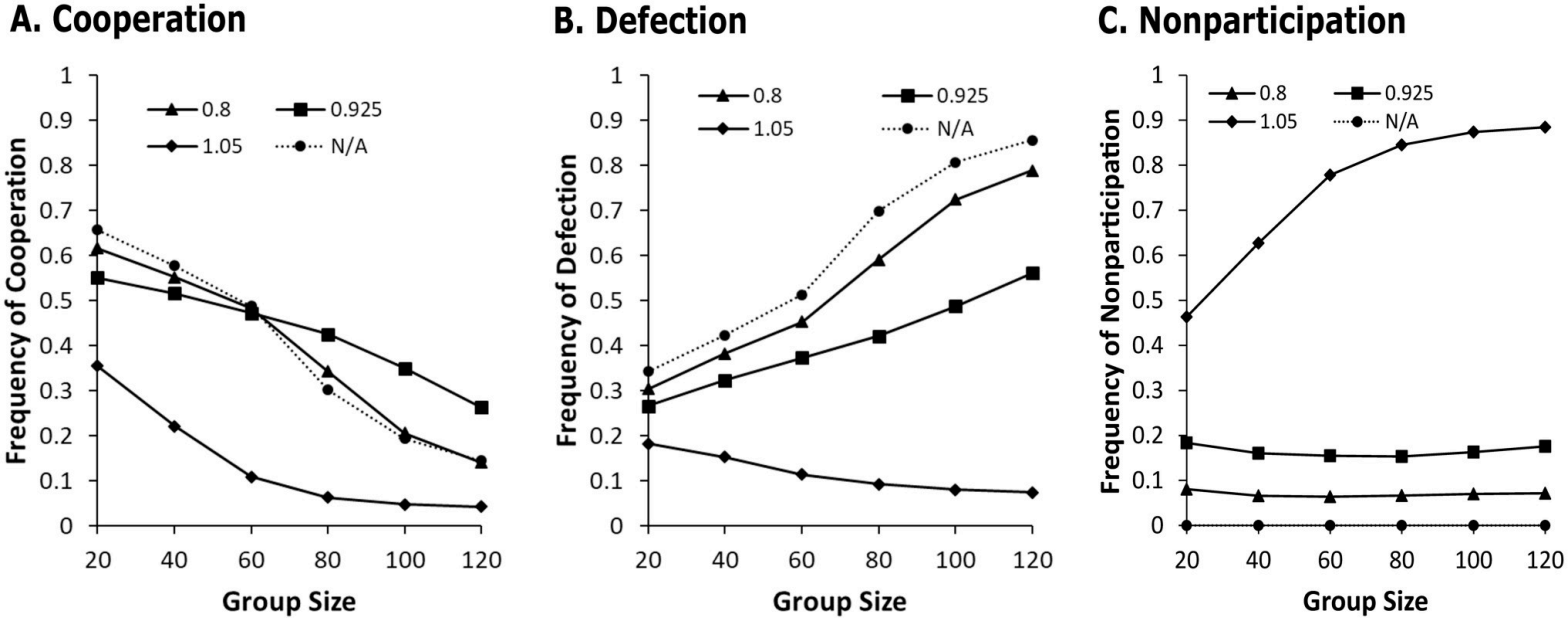
The long run average frequencies of participation (cooperation and defection) and nonparticipation. (A) Cooperation (including contributors and punishers); (B) Defection; and (C) Nonparticipation. The dotted lines indicate the long run results of the compulsory participation scenario, and the solid lines the long run results of the voluntary participation scenarios. Three types of markers (triangle, square, and diamond) of the solid lines represent the low level of nonparticipants’ payoff (0.8), the moderate (0.925), and the high (1.05), respectively. N/A signifies compulsory participation. The data used in this figure are provided in S1 Data.

Fig 2 helps us to take a close look at population dynamics behind the asymmetric effects of voluntary participation on the evolution of cooperation. Fig 2 shows how the long run average frequencies of defection and nonparticipation in addition to cooperation. Based on the results shown in Fig 1, we selected three representative levels of nonparticipants’ payoffs (i.e., 0.80, 0.925, and 1.05) that captures the concave relationship between nonparticipants’ payoff and cooperation. For convenience, we shall call 0.80 the low payoff, 0.925 the moderate payoff, and 1.05 the high payoff.

Fig 2A shows that compared to compulsory participation (dotted line), the low payoff (triangle marker) makes little or no difference for the long run average of cooperation across group sizes. Compared to compulsory participation, the low payoff contributes to reducing the frequency of defection (Fig 2B). The decreased defection coincides with an increase in nonparticipants (Fig 2C) so that the frequency of cooperation does not change significantly. The high payoff to nonparticipants (diamond marker) has an obviously negative effect on cooperation across group sizes (Fig 2A), while reducing the number of defectors remarkably (Fig 2B). The reason why the decreased defection does not lead to an increase in cooperation is that the nonparticipants’ payoff is high enough to reduce the fitness of participants in their joint project. Such a great deal of nonparticipation is shown in Fig 2C.

An interesting finding is that the moderate payoff (0.925) has an asymmetric effect on cooperation across group sizes. To put it another way, as shown in Fig 2A, the moderate payoff (square marker) negatively affects the frequency of cooperation for relatively small groups (*n* = 20, 40) but positively for relatively large groups (e.g., *n* = 80, 100, *and* 120). As the group size gets larger, the frequency of defection at the moderate payoff becomes lower than that in the compulsory participation situation by greater margin. This is represented by an increase in the vertical distance between square and circle markers in Fig 2B. However, the frequency of nonparticipation is almost constant (approximately 0.2) regardless of the group size (Fig 2C). These trends help us to arithmetically understand that the frequencies of cooperation for relatively large groups are higher at the moderate payoff than observed in the compulsory participation situation. This result suggests that the moderate payoff becomes more effective in enhancing levels of cooperation as the group size gets larger.

Why does the moderate payoff increase levels of cooperation only in relatively large groups so that the inverted U-shaped curves (Fig 1) emerge in those groups? One plausible explanation is that compared to small groups, large groups are more likely to sustain sufficient differences between groups in the frequency of defectors and nonparticipants (conversely, the frequency of cooperators). Maintaining substantial between-group variation in the frequency enables group-selection force to have a stronger effect on levels of cooperation (Boyd et al. 2003). For instance, when a group with 20% cooperators of its population encounters another group with 80% cooperators, both groups will evolve towards having 80% cooperators because the latter takes over the former. However, when a group with 20% cooperators encounters another group with 30% cooperators, group selection will lead both groups to have only 30% cooperators.

We speculate that enough variation between groups in the frequency of cooperators could be maintained for a longer period of time in larger groups. This is because compared to small groups, a higher payoff behavior diffuses more slowly within large groups through the individual-level payoff biased imitation. In summary, such a relatively slower spread of a more lucrative behavior within large groups helps maintain enough variation between groups, thereby increasing the positive effect of *group-selection* force on levels of cooperation.

We tested the sensitivity of our model to variations in two stochastic parameters, rate of mixing between groups (*m*), and mutation rate (*μ*), and a payoff parameter, the cost of being punished (*p*). Overall, the observed effects of such parameters on levels of cooperation are similar to those presented in Boyd et al. (2003)’s compulsory participation model: 1) decreasing the mixing rate increases the long run average frequency of cooperation while increasing the rate reduces the frequency of cooperation; 2) decreasing the mutation rate remarkably increases the frequency of cooperation while increasing the rate, on average, decreases the frequency; 3) the frequency of cooperation significantly decreases when punishment is absent or inflict less cost on defectors. More details on the sensitivity analysis are provided in S1 File.

## Discussion

Our model analysis focused on examining combined effects of three evolutionary forces linked to voluntary participation and intergroup competition, namely *defector-decreasing*, *punisher-decreasing*, and *group-selection* forces, on the levels of cooperation. *Defector-decreasing* and *punisher-decreasing* forces are associated with nonparticipation and derive from payoff-biased imitation at the individual level, while *group-selection* force comes from interactions between groups. Our analysis was motivated by a research gap that it remains unclear under what conditions their combined effects can enhance levels of cooperation (including non-punishing and punishing cooperation) in the long run.

*Defector-decreasing* force signifies that allowing nonparticipation could lead defectors to opt out of a joint project. To the extent this is the case, the cost of punishing, which is proportional to the number of defectors, goes down. Such a payoff advantage of punishers will likely increase the number of punishers. Hence, an additional decrease in defectors could occur. *Punisher-decreasing* force means that allowing nonparticipation could lead punishers to opt out of a joint project. To the extent this is the case, the cost of being punished, which is proportional to the number of punishers, goes down. Such a payoff advantage of defectors will likely increase the number of defectors, and thus leads to the payoff disadvantage of punishers. Hence, an additional decrease in punishers could occur. With g*roup-selection* force, cooperative groups are more likely to survive selection pressures and pass on group-level properties that benefit groups, which means the spread of punishers and the reduction of defectors. Although these three forces do not constitute an exhaustive list of evolutionary forces, they capture general features found in many real-world situations, namely individuals can freely decide whether or not to participate in a joint work and their payoff is affected by both individual- and group-level properties.

Our model analysis shows that the option of nonparticipation can have asymmetric effects on cooperation across different levels of nonparticipant payoffs and group sizes. The simulation results of the nonparticipants’ low payoff (see triangle markers in Fig 2) show that voluntary participation has little or no effect on levels of cooperation compared to compulsory participation. If nonparticipants’ payoff is too low, it rarely incentivizes defectors and punishers opt out of public goods provision. Thus, neither *defector-decreasing* nor *punisher-decreasing* force can be activated sufficiently, while *group-selection* force is in effect. Hence, the results of evolution of altruistic punishment generated from voluntary and compulsory participation scenarios are very similar.

The simulation results of the nonparticipants’ high payoff (see diamond makers in Fig 2) report a dramatic drop in levels of cooperation in comparison to the results of compulsory participation. If nonparticipants’ payoff is too high, both defectors and punishers will likely become nonparticipants very quickly and simultaneously. This means that both *defector-*and *punisher-decreasing* forces are fully activated so that the number of participants (including cooperators and defectors) diminishes substantially. This leads to a decrease in the payoffs to participants, which in turn leads to an additional reduction in the number of participants. Given the vicious cycle due to individual payoff-biased imitation, *group-selection* force is not at play in enhancing levels of cooperation because nonparticipation outcompetes cooperative behaviors.

**Fig 3:**
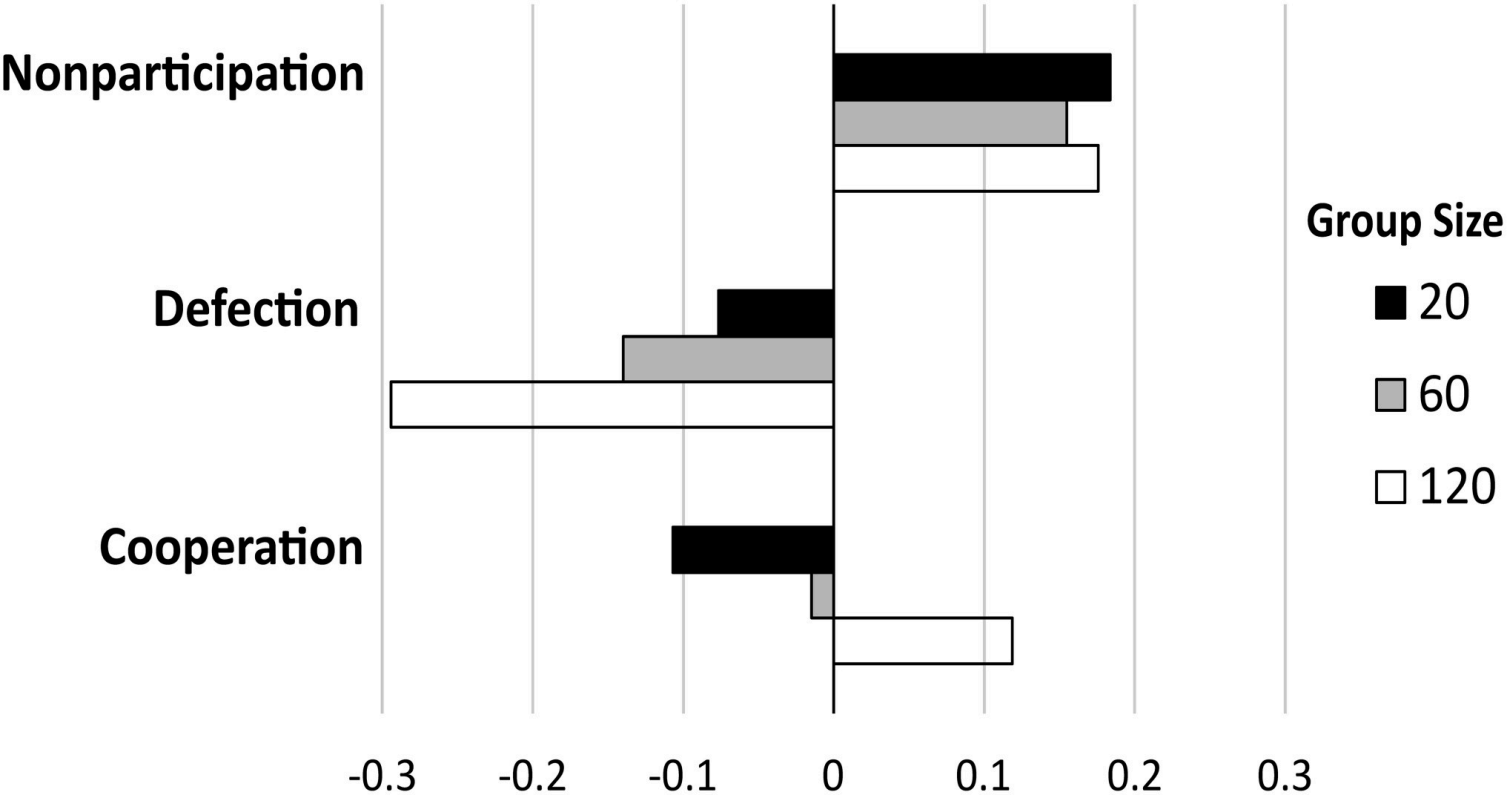
Differences in the long run average frequencies of behaviors across group sizes. Given the moderate level of nonparticipants’ payoff (0.925), each bar represents the difference calculated by subtracting the frequency of each behavior in the compulsory participation scenario from that in the voluntary participation scenario. Three different colors of bars represent group sizes. For instance, the bottom white bar shows that voluntary participation makes the long run average frequency of cooperation higher than compulsory participation, given that group size is large (*n* = 120) and the nonparticipants’ payoff is moderate. The data used in this figure are provided in S2 Data.

In contrast, the simulation results under moderate nonparticipation payoffs indicate that the effects of voluntary participation on cooperation vary with group size. This group-size effect is an unanticipated outcome while we expected the asymmetric effect of nonparticipants’ payoff on levels of cooperation. The group-size asymmetric effect of voluntary participation on cooperation is visualized as the opposite directions of the three bars for cooperation in Fig 3. A black bar for cooperation indicates a negative effect of voluntary participation on cooperation in small groups (*n* = 20). This is based on the observation from Fig 2A that the frequency of cooperation in the small groups becomes lower at the moderate level of nonparticipants’ payoff than the frequency of cooperation in the compulsory participation scenario. Similarly, a white bar for cooperation in Fig 3 shows a positive effect of voluntary participation on cooperation in large groups (*n* = 120), and a gray bar indicates that there is little or no change in the frequency of cooperation in intermediate groups (*n* = 60).

This asymmetric effect associated with group size observed at the moderate payoffs can be explained by the three evolutionary forces described above. Note that *defector-decreasing* and *group-selection* forces contribute to reducing the number of defectors, whereas *punisher-decreasing* force helps decrease the number of punishers. In the case of *n* = 120 (white bars), a significant decrease in defection is accompanied by an increase in cooperation. This indicates that an aggregate of *defector-decreasing* and *group-selection* forces outcompetes the *punisher-decreasing* force in the long run. In other words, the joint effect of the first two forces is so strong that a decrease in the cost of punishing, *k*(1 − *x* − *z*), can be sufficient to contribute to the spread of punishers (thus, a decrease in the number of defectors) despite *punisher-decreasing* force. The case of *n* = 60 (gray bars) reports that nonparticipation is effective in suppressing the spread of defectors, but not that much effective in enhancing levels of cooperation. This means that the joint effect of *defector-decreasing* and *group-selection* forces is somewhat strong, but not enough to overwhelm *punisher-decreasing* force. Lastly, the case of *n* = 20 (black bars) shows a reduction in both defection and cooperation. The directions of the defection and cooperation bars are all negative, and their lengths are similar. This suggests that the joint effect of *defector-decreasing* and *group-selection* forces is barely keeping with the *punisher-decreasing* force in the long run.

## Conclusion

In this study, we set out to address the question of how voluntary participation and group selection jointly affect the evolution of altruistic punishment. In approaching this aim, we created and analyzed a multi-level selection model of public goods provision structured by three evolutionary forces (*defector-decreasing*, *punisher-decreasing*, and *group-selection* forces) linked to voluntary participation and group selection. We have shown that the effects of voluntary participation on cooperation vary with nonparticipants’ payoff and group size. Compared to compulsory participation, voluntary participation can achieve higher frequencies of cooperation, but this happens only within a certain range of nonparticipants’ payoff. As their payoff exceeds a certain point, the frequency of cooperation sharply drops in voluntary participation scenarios. However, such an asymmetric effect is observed only in relatively large groups. In small groups, no range of nonparticipants’ payoff was associated with enhanced frequency of cooperation. This means that there is another asymmetric effect of voluntary participation with respect to group size.

Our results have some broader implications. People often and voluntarily withdraw from an objectional state of affair instead of staying put to make their voice heard. In market contexts, customers do not simply buy goods and services in decline, and some members do not participate in group activities when group decision or performance is disappointing. Since human behavior of that kind was called *exit* by Hirschman [36], a variety of social science disciplines have studied the effects of *exit* on group performances in different contexts, such as prisoners’ dilemma games [16, 17, 24], irrigation systems [32], group formation [37, 38], group associations [39], public services [40], democratization and economic performance [41], and exit-based democracy [42]. Interestingly, a rich body of studies on the evolution of human cooperation have been interested in ‘voluntary participation’ in public goods games that guarantees an opportunity for individuals to exercise the *exit* option. This common denominator may allow us to gain fruitful insights from prior studies of exit to enrich our understanding of the effects of voluntary participation on the evolution of human cooperation.

Future models of voluntary participation may consider the possibility that individuals do not participate in a group’s joint project but instead join another group to participate in the group’s project [37, 38]. Future studies may also include a parameter that affect the fitness of participants in a joint endeavor, depending on the experimental evidence that cooperators are more likely to play a prisoners’ dilemma game than defectors [16, 17]. In line with the existing studies of voluntary participation, we assumed that nonparticipants cannot contribute to producing a local public good. However, in a more contemporary rural context, outmigrants who voluntarily leave their groups can also contribute part of remittance to providing local public goods in places of migrant origination [43]. Lastly, nonparticipants’ payoffs could be treated not as an exogenously set parameter, but rather, as endogenously changing values if their payoffs depend on rival consumption of private goods.

## Supporting information

S1 Text ODD protocol. We presented the ODD protocol to describe the current agent-based model.
(DOCX)

S1 Data Simulation results. We presented the simulation data for Figs 1 and 2.
(XLSX)

S2 Data Simulation results. We presented the simulation data for Fig 3.
(XLSX)

S1 Code NetLogo 6.1.1. We presented the code of the current agent-based model.
(NLOGO)

S1 File Sensitivity analysis. We presented the results of sensitivity analyses of three key parameters.
(DOCX)
