# Supplementary material for: Joint effects of voluntary participation and group selection on the evolution of altruistic punishment
Source: PLoS One. 2022 May 4;17(5):e0268019. doi: 10.1371/journal.pone.0268019 (PMC9067692; doi:10.1371/journal.pone.0268019)
Supplement: S1 Text — We presented the ODD protocol to describe the current agent-based model. (DOCX) [file pone.0268019.s001.docx]

**Supporting Information**

**Model Description: ODD Protocol**

The model description follows the ODD (Overview, Design concepts, Details) protocol for describing individual- and agent-based models [1] and consists of seven elements. The first three elements provide an overview, the fourth element explains general concepts underlying the model’s design, and the remaining three elements provide details. Additionally, details of the software implementation are presented.

**1. Purpose**

The purpose of this model is to investigate joint effects of voluntary participation and group selection on the evolution of altruistic punishment. In our model, nonparticipation is defined as *neither producing a local public good nor consuming the public good produced by others*. Hence, nonparticipation conceptually differs from defection which is defined in many public goods games (or prisoners’ dilemma games) as consuming a public good produced by others without contributing to the provision of the public good. On the other hand, a logic of group selection is that more cooperative groups are more likely to survive selection pressures such as inter-group interactions (e.g., warfare) and success/failure in sustaining natural resources (e.g., common-pool resources). These two mechanisms are incorporated into a multilevel selection model to examine how exogenous changes in nonparticipants’ payoff affect the evolution of altruistic punishment across group sizes

**2. State variables and scales**

An artificial society of this model consists of 128 groups. Group population size $(n)$ and nonparticipants’ payoff $(\Omega)$ vary in the simulations. Each group provides a local public good in two different situations: compulsory and voluntary participation in public goods provision. In a compulsory participation scenario, there are three behavioral types of individual members within a group: defection (defector), cooperation without punishment (contributor), and cooperation with punishment (punisher). With a small probability individuals encounter each other and imitate higher payoff individuals. Besides the payoff-biased imitation among individuals, groups with more cooperators (including contributors and punishers) are more likely to take over the other groups with fewer cooperators. In voluntary participation scenarios, nonparticipation is added to the three behavioral types above, i.e., defection, contribution, and punishment. Note that like the compulsory participation scenario, these four types of behaviors interact and propagate through the two mechanisms, such as individual payoff-biased imitation and cultural group selection.

**3. Process overview and scheduling**

Initially, one group consists of all punishers and the other 127 groups consist of all defectors. Based on the initial set-up, five sequential events occur in each time period: 1) cooperators (contributors plus punishers) within a group contribute to a local public good with probability $1-e$ and erroneously defect with probability $e$, while such an error does not occur to defectors; 2) punishers within a group reduce the payoff of each defector within the group; 3) payoff biased imitation occurs among group members, which means that members within a group encounter another member from their own group with probability $1-m$ and a member from another randomly chosen group with probability $m$ (see 7. Submodels for more details on individual-level payoff biased imitation); 4) cultural group selection occurs among groups, which means that one group with more cooperators to take over one of the other groups which is randomly paired with the group with probability $s$ (see 7. Submodels for more details on group selection); 5) a small change of mutation occurs to each member with probability $\mu$ (e.g., a cooperator switches to a defector). Note that, with the five stages unchanged in voluntary participation scenarios, the option of nonparticipation is initially introduced to group members via mutation, i.e., participants in public goods provision flips to nonparticipants with probability $\mu$.

**4. Design concepts**

- *Emergence*. Emergence of cooperators (contributors plus punishers) who produce a local public good.
- *Adaptation*. Groups adapt their composition of members through multilevel selection mechanisms, such as individual payoff-biased imitation, mutation, and cultural group selection.
- *Fitness*. The fitness of a group for cultural group selection is associated with the frequency of cooperation.
- *Interaction*. Individuals interact through payoff biased imitation, and groups interact via cultural group selection.
- *Stochasticity*. (1) Probability of mutation: individuals of each type spontaneously switch into one of the other types by a small probability; (2) Probability of mixing between groups: each member within a group encounters a member from a randomly chosen group by a small probability; and (3) Probability of cultural group selection: groups are randomly paired to take over other groups by a small probability

**5. Initialization**

Simulations start with one group consisting of only punishing cooperators and the other 127 groups consisting of only defectors.

**6. Input**

Boyd et al. [2] do not consider nonparticipation in public goods provision but incorporate individual payoff-biased imitation and cultural group selection. Our model aims to compare the results of compare compulsory and voluntary participation scenarios. To make a *ceteris paribus* setting for the cross-scenario analysis, all default values of the parameters (except for $n$ and $\Omega$) are set to be the same as in Boyd et al. [2].

Default parameter values

- $N=128$; Number of groups
- $n=20, 40, 60, 80, 100, 120$; Number of members in a group
- $b=0.5$; Benefit from a local public good if every group member cooperates
- $c=0.2$; Cost of cooperation
- $p=0.8$; Cost of being punished
- $k=0.2$; Cost of punishing (Boyd et al. 2003)
- $m=0.01$; Rate of mixing between groups (for individual imitation)
- $\mu=0.01$; Mutation rate
- $s=0.015$; Rate of group pairing (for cultural group selection)
- $e=0.02$; Erroneous defection rate
- $\Omega=0.80 to 1.30 in 0.05 increments$; nonparticipants’ payoff

**7. Submodels**

Here, I describe two key simulation scenarios: a compulsory participation scenario in which group members have to participate in public goods provision; and voluntary participation scenarios in which group members are allowed to withdraw from public goods provision.

1. Compulsory participation scenario ([2])

- The payoff for a contributor is $1+bx-c$, where $x$ is the fraction of cooperators in a group.
- The payoff for a defector is $1+bx-py$, where $y$ is the fraction of punishers in a group.
- The payoff for a punisher is $1+bx-c-k(1-x)$

*Payoff-biased individual imitation*Members within a group encounter another member from their own group with probability $1-m$ and a member from another randomly chosen group with probability $m (=0.01)$. A member $i$ encounters a member $j$ and imitates $j$ with probability $\frac{w_{j}}{(w_{j}+w_{i})}$ , where $w_{q}$ is the payoff of member $q$ in the game, including the costs of punishing and being punished. This individual imitation leads to not only the spread of higher payoff behaviors within groups, but also diffusion of the behaviors between groups with probability $m$.

*Cultural group selection*In each time step, group selection occurs. Each group is randomly paired with one of the other groups with probability $s (=0.015)$. Their interaction leads one group to take over another group. The probability that group $i$ takes over group $j$ is $0.5\{1+\left( x_{i}-x_{j} \right)\}$, where $x_{g}$ is the frequency of cooperators (contributors plus punishers) in group $g$. This means that the group with more cooperators is more likely to take over another group with fewer cooperators.

2. Voluntary participation scenarios

- The payoff for a contributor is the same as that in the compulsory participation scenario.
- The payoff for a defector is the same as that in the compulsory participation scenario.
- The payoff for a punisher is $1+bx-c-k(1-x-z)$, where $z$ is the frequency of nonparticipants. Nonparticipants’ payoff $(\Omega)$ is exogenously given to group members while the payoffs for participants in public goods provision vary with endogenous population dynamics. In each simulation of voluntary participation scenarios, nonparticipants’ payoff is set to be one of a contributor’s possible payoffs ranging from $1-c$ (when $x=0$) to $1+b-c$ (when $x=1$). Once one of the nonparticipants’ payoffs is given to a simulation, it is constant throughout the simulation.

*Payoff-biased individual imitation*This is the same as in the compulsory participation scenario.

*Cultural group selection*This is the same as in the compulsory participation scenario.

**8. Model implementation**

Our model was implemented in NetLogo 6.1.1. To develop our multilevel selection model, we modified a model which was developed by Dr. Marco Janssen [3] to replicate Boyd et al. [2]’s model. We followed Boyd et al. [2] both in terms of a span of simulation time (time step = 1 year) and a way of calculating the long run average frequency of cooperation. Each result is the average of 100 simulations. For each simulation, we reported the average frequency of cooperation over the last 1,000 time periods of a 2000 time-period simulation. The results are plotted in Fig. 1.

**References**

1. Grimm V, Berger U, Bastiansen F, Eliassen S, Ginot V, Giske J, et al. A standard protocol for describing individual-based and agent-based models. Ecol Modell. 2006;198: 115–126. doi:10.1016/j.ecolmodel.2006.04.023

2. Boyd R, Gintis H, Bowles S, Richerson PJ. The evolution of altruistic punishment. Proc Natl Acad Sci. 2003;100: 3531–3535. doi:10.1073/pnas.0630443100

3. Janssen M. Evolution of altruistic punishment. CoMSES Computational Model Library; 2019. Available: https://www.comses.net/codebases/2223/releases/1.2.0/
