# Supplementary material for: Joint effects of voluntary participation and group selection on the evolution of altruistic punishment
Source: PLoS One. 2022 May 4;17(5):e0268019. doi: 10.1371/journal.pone.0268019 (PMC9067692; doi:10.1371/journal.pone.0268019)
Supplement: S1 File — We presented the results of sensitivity analyses of three key parameters. (DOCX) [file pone.0268019.s005.docx]

**Supplementary Information 5:**

**Sensitivity Analysis**

We tested the sensitivity of our model to variations in three parameters: 1) rates of mixing between groups $(m)$, 2) mutation rates $(\mu)$, and 3) the costs of being punished $(p)$. For the sensitivity analyses, we selected four group sizes including the minimum $(n=20)$, the maximum $(n=120)$, and the two group sizes $\left( n=60 and 80 \right)$ between them because in the main text, they showed how the effect of voluntary participation on levels of cooperation varies with group sizes. We also selected four levels of nonparticipants’ payoff $\left( \Omega=0.85, 0.95, 1.05, and 1.15 \right)$ because they showed the culvilinear (or inverted U-shaped) relationship of nonparticipants’ payoffs with levels of cooperation in our main analyses.

Note that like our results shown in the main text, the sensitivity analysis results below are also the long run average frequencies of behavioral types over the last 1,000 time periods of 100 simulations. Behavioral types in our multi-selection model are: 1) defectors who do not contribute to the public good but exploit the contributions of the other participants; 2) contributors who contribute but do not punish the defectors; 3) punishers who not only contribute but also punish the defectors; and 4) nonparticipants who neither produce a public good nor consume the public good produced by cooperators. Recall that the term ‘cooperators’ in this study represents a group of both contributors and punishers.

**1. Sensitivity analysis: rate of mixing between groups** $\boldsymbol{(}\boldsymbol{m)}$

We investigated how the long run average frequency of cooperation varies with rates of mixing between groups $(m=0.002, 0.01, and 0.05)$. In Fig S1, we plotted the results of our sensitivity analyses across group sizes (Panels A to D) with a range of nonparticipants’ payoff (0.85 to 1.15 on the X-axis).


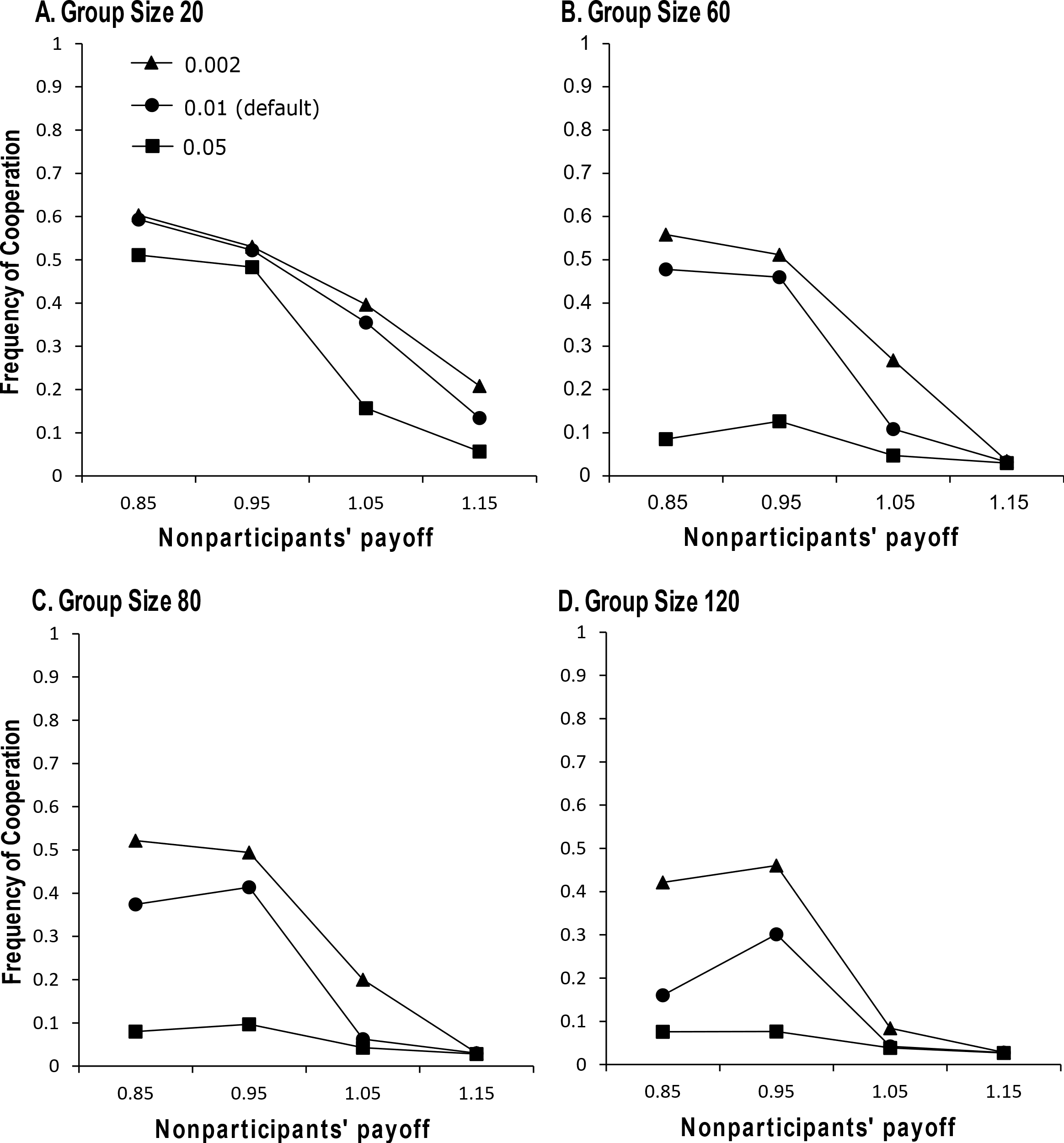


Fig S1. The rate of mixing between groups significantly affects the evolution of cooperation (contributors and punishers). We considered three mixing rates $(m=0.002, 0.01, and 0.05)$ across group sizes (panels A to D) with a range of nonparticipants’ payoff (X-axis). Three key findings are: 1) increasing the rate of mixing reduces the long run frequency of cooperation; 2) two asymmetric effects of nonparticipants’ payoff on the long run average frequency of cooperation are maintained unless mixing rate is too high; and 3) at the highest rate of mixing (square markers), cooperation falls precipitously in relatively large groups regardless of nonparticipants’ payoff levels.

A key finding is that increasing the rate of mixing reduces the long run frequency of cooperation (see square markers in Fig S1). We also found that two asymmetric effects of voluntary participation on the long run average frequency of cooperation are maintained unless the mixing rate is too high. Recall that the asymmetric effects are associated with group sizes and nonparticipants’ payoff levels: voluntary participation negatively affects the evolution of cooperation in relatively small groups; and it has positive impacts in larger groups within only a limited rage of nonparticipants’ payoff. Lastly, we found that cooperation falls precipitously in relatively large groups regardless of levels of nonparticipants’ payoff at the highest rate of mixing (see square markers in Fig S1).

A fundamental mechanism behind these results is as follows. In our model, the mixing rate is defined as the probability $(m)$ that members in a group encounter a member from another randomly chosen group; conversely, they encounter another member from their own group with probability $(1-m)$. The imitation of high payoff individuals will then occur between them. As the mixing rate gets higher, higher payoff behaviors will likely diffuse from one group to another more quickly. If nonparticipants’ payoff is too small, defection will spread between groups more quickly than cooperative behaviors because cooperation has no individual level benefits. But if nonparticipants’ payoff gets larger, nonparticipation will diffuse between groups more rapidly than the other strategies including defection. Such a “migration-like process” (Boyd et al. 2003, 3532) will likely decrease between-group differences in the frequency of cooperation.

In our model, the decrease in the frequency variation between groups has a negative effect on levels of cooperation. This is because a sufficient level of the between-group difference is a necessary condition for *group-selection* force to become more effective in enhancing levels of cooperation. The reason is as follows. According to the payoff-biased, individual-level imitation, defectors cannot spread easily in some groups where punishers are common while they do well in the other groups where punishers are rare. If the rate of mixing between groups is relatively low, such a “selection-like process” (Boyd et al. 2003, 3532) within groups is more likely to occur than the “migration-like process” between groups. As the likelihood of the selection-like process increases, it is more likely that a sufficient level of variation between groups in the frequency of cooperation is maintained. Given the sufficient variation, *group-selection* force can help cooperation spread between groups. However, if the mixing rate is too high, the selection-like process cannot maintain a sufficient level of frequency variation between groups. Given the lack of variation, the positive effect of *group-selection* force on levels of cooperation becomes weaker.

Fig S2 depicts the resulting outcomes across mixing rates (lower row on the X-axis) and nonparticipants’ payoffs (upper row on the X-axis). As the mixing rate gets higher, the long run frequency of cooperation (black cylinder) becomes lower at each level of nonparticipants’ payoff whereas the frequency of defection (gray cylinder) or nonparticipants (white cylinder) gets higher: the frequency of defection is higher than that of nonparticipation at the nonparticipants’ low payoff while it is dramatically reversed at their high payoff because of the payoff-biased imitation.


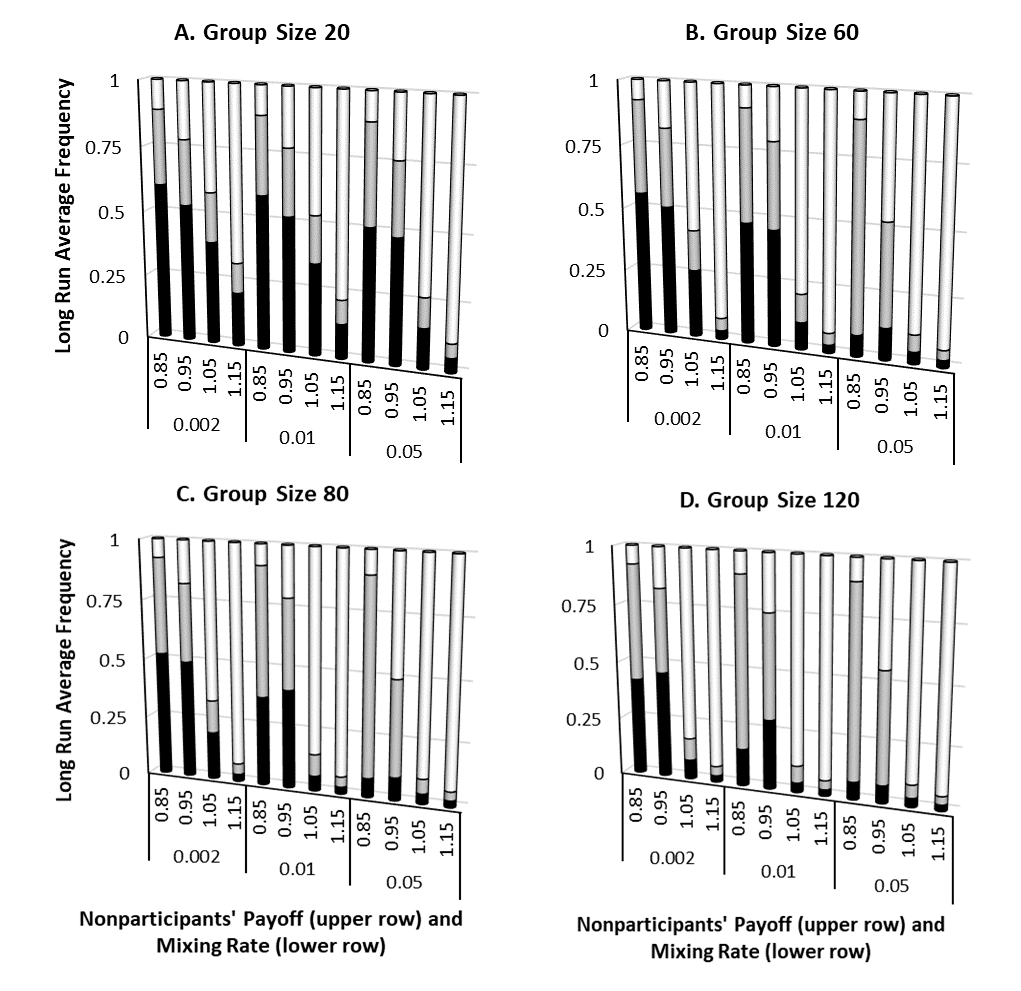


Fig S2. Variations in the long run average frequencies of behaviroal types across nonparticipants’ payoffs (upper row on the X-axis) and rates of mixing between groups (lower row on the X-axis). Panels A to D depict the variations in the frequencies for different sizes of groups. The black cylinder indicates the frequency of cooperation (contributors and punishers), the gray the frequency of defection, and the white the frequency of nonparticipation.

**2. Sensitivity analysis: mutation rate** $\boldsymbol{(}\boldsymbol{\mu)}$

Our model assumes that a small change of mutation occurs with probability $(\mu)$. For instance, defectors can flip to contributors and participants in public goods provision (i.e., contributors, punishers, and defectors) can also flip to nonparticipants. Note that an initial introduction of nonparticipants in our model depends solely upon the stochastic process of mutation. For example, nonparticipants will not exist in our model if $\mu=0$. We explored how the long run average frequency of cooperation changes with three levels of mutation rate $(\mu=0.002, 0.01, and 0.05)$. Fig S3 depicts the sensitivity analysis results.


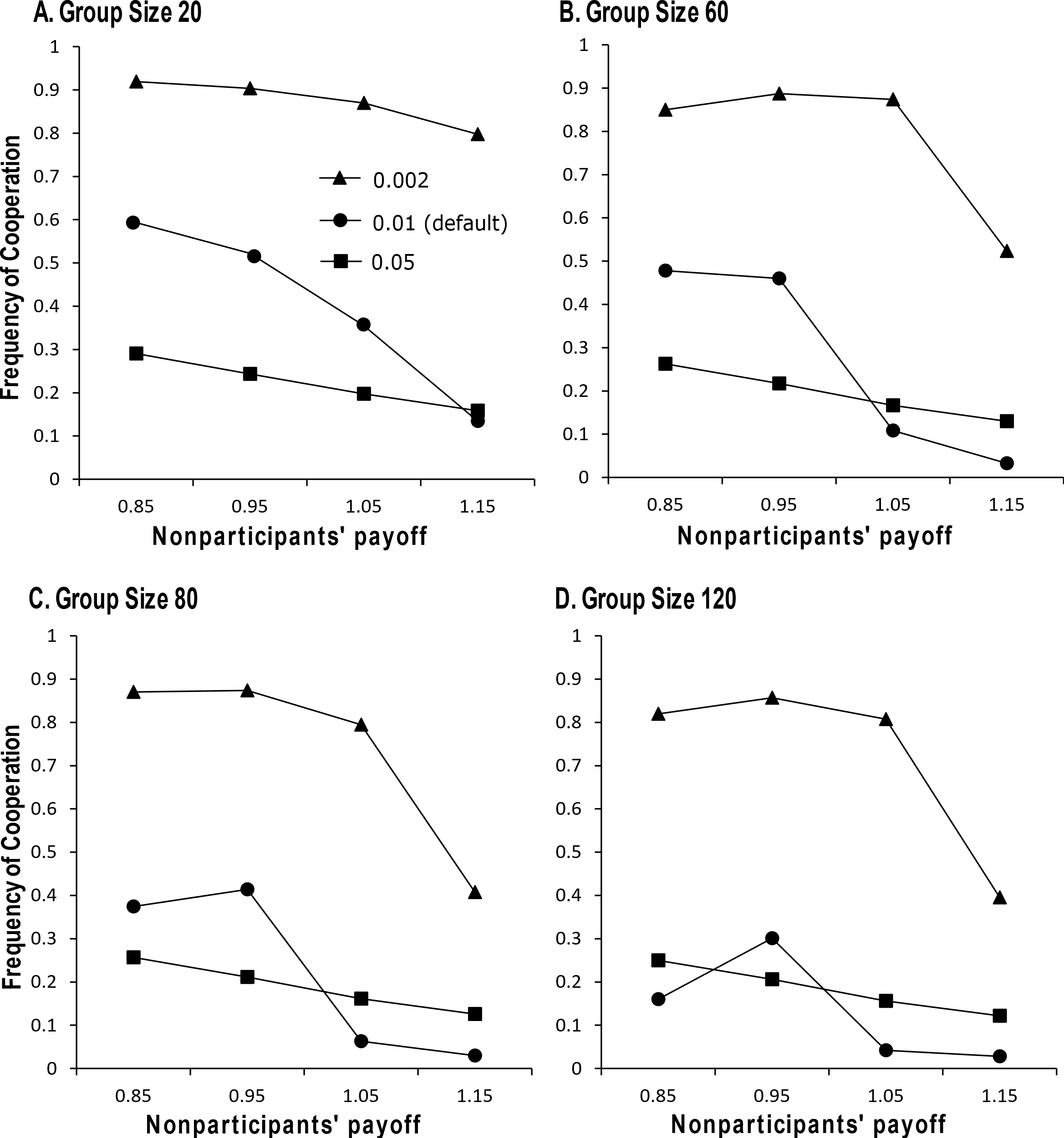


Fig S3. The mutation rate significantly affects the evolution of cooperation (contributors and punishers). We considered three mutation rates $(\mu=0.002, 0.01, and 0.05)$ across group sizes (panels A to D) with a range of nonparticipants’ payoff (X-axis). Two key findings are: 1) decreasing the mutation rate to 0.002 (triangle markers) significantly increases the long run average frequency of cooperation; and 2) at the highest rate of mutation (square markers), the frequency of cooperation remains low at all nonparticipants’ payoff levels regardless of group sizes, and the frequency decreases almost linearly with nonparticipants’ payoff.

Fig S3 shows that decreasing the mutation rate to 0.002 (triangle markers) significantly increases the long run average frequency of cooperation. If the mutation rate is too small, the number of nonparticipants can hardly increase in our model because their first appearance depends only upon mutation. With nonparticipants rare, voluntary participation mechanism cannot be activated sufficiently in our model. Hence, no significant differences will be observed in the results of voluntary and compulsory participation scenarios. Indeed, such a dramatic increase in levels of cooperation (see triangle markers) is in line with the results of compulsory participation scenarios reported by Boyd et al. (2003). Additionally, when the mutation is too low, it is plausible to think that nonparticipation can survive only if the payoff of nonparticipants is very high; otherwise, nonparticipation will be defeated by the other strategies given that it rarely appears due to the small mutation rate. Our simulation results confirm this prediction (see white bars in Panels B, C, and D of Fig S4, at $\mu=0.002$).


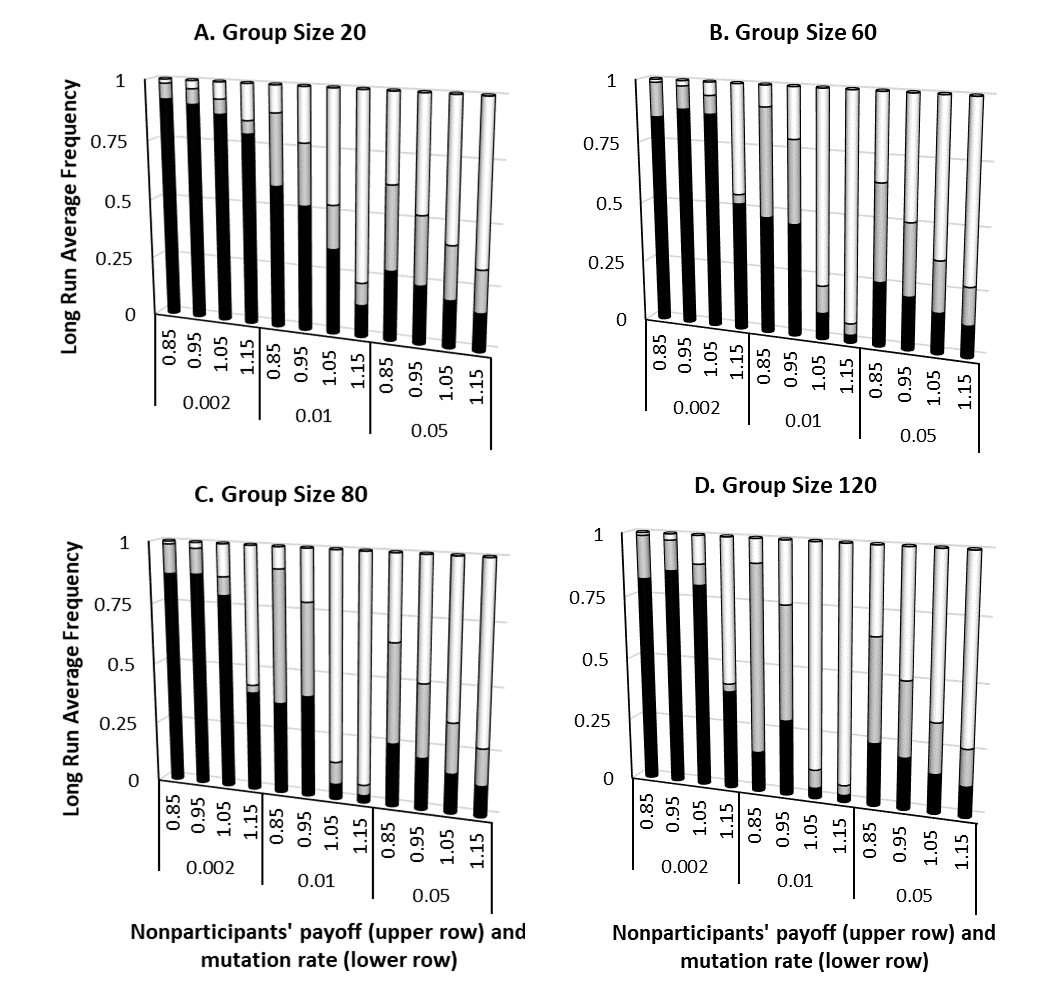


Fig S4. Variations in the long run average frequencies of strategies across nonparticipants’ payoffs (upper row on the X-axis) and rates of mutation rates (lower row on the X-axis). Panels A to D depict the variations in the frequencies for different sizes of groups. The black cylinder indicates the frequency of cooperation (contributors and punishers), the gray the frequency of defection, and the white the frequency of nonparticipation.

At the highest mutation rate (square markers in Fig S3), on the other hand, the long run frequency of cooperation decreases linearly as nonparticipants’ payoff becomes larger. And the frequency remains lower than 0.3 at any level of nonparticipants’ payoff. These stable patterns are observed regardless of group sizes. It is difficult to fully understand why such patterns emerge, since they result from the multiple stochastic processes such as mutation, imitation, and group selection. Nevertheless, one of the useful clues for explaining the stable patterns is found in the study of the effects of mutation rates on evolutionary dynamics in public goods games [2]. For high mutation rates and large groups (i.e., $\mu\times n\gg1)$, all strategic types always exist in the groups. This could lead to smaller between-group differences in the frequencies of defectors and nonparticipants. Hence, it is plausible to hypothesize that, when the mutation rate is high, the other stochastic processes (particularly group selection) are less likely to help enhance levels of cooperation.

**3. Sensitivity analysis: the cost of being punished** $\boldsymbol{(p}\boldsymbol{)}$

We tested the sensitivity of our model to variations in the cost of being punished $(p=0.0, 0.4, and 0.8)$. Note that $p=0.0$ represents the absence of punishment which means that the cost of punishing is also zero $(k=0.0)$. In the absence of punishment, defectors’ payoff is always greater than cooperators’ because defectors do not bear both the cost of being punished and the cost of producing a local public good. Such a payoff advantage of defectors will likely lead defection to spread more rapidly, thereby contributing to a dramatic reduction in cooperator. This is true when nonparticipants’ payoff is low enough for defection to be more lucrative than nonparticipation. Fig S5 shows that the long run average frequency of cooperation falls precipitously (square markers) at the low and moderate payoffs of nonparticipants (e.g., 0.85 and 0.95) regardless of group sizes.


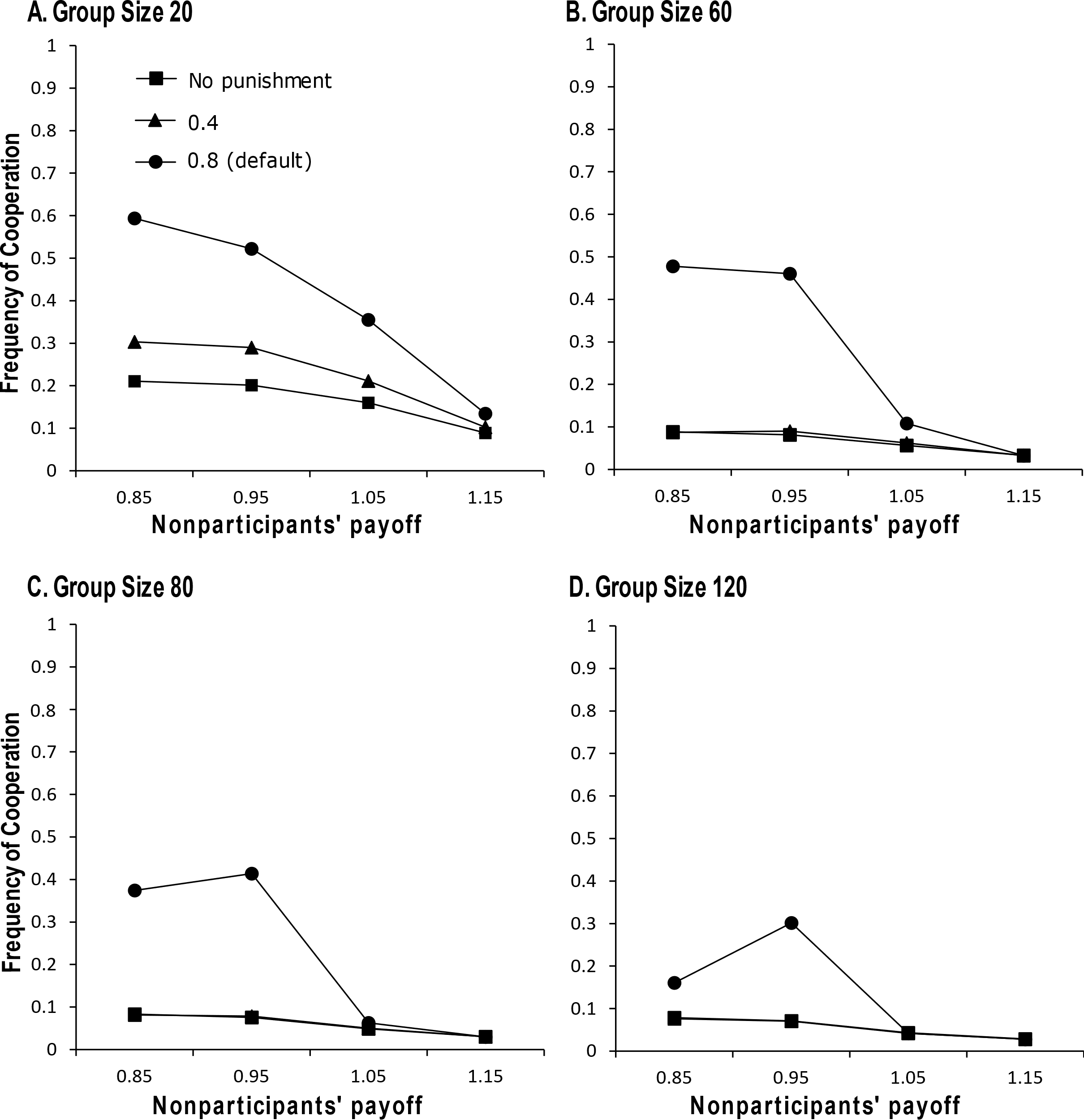


Fig S5. The cost of being punished significantly affects the evolution of cooperation (contributors and punishers). We considered three costs of being punished $(p=0.0, 0.4, and 0.8)$ across group sizes (panels A to D) with a range of nonparticipants’ payoff (X-axis). Three key findings are: 1) without punishment (square markers; $p=0.0$), the frequency of cooperation remains very low regardless of group sizes and nonparticipants’ payoff; 2) decreasing the cost of being punished (triangle markers) reduces the long run average levels of cooperation; and 3) the results from the absence of punishment are almost the same as those from the low cost of being punished.

As shown in Fig S6, the massive reduction in cooperation coincides with a substantial increase in defectors rather than nonparticipants (compare the first two gray and black bars at $p=0.0$). This implies that neither *defector-* nor *punisher-decreasing* force is activated because the nonparticipants’ payoffs (0.85 and 0.95) is not sufficiently high. Given the lack of the two forces, the evolution of cooperation depends heavily on the *group-selection* force. Hence, the simulation results of voluntary participation are in line with those of compulsory participation which are depicted in Fig 2a in Boyd et al. (2003).


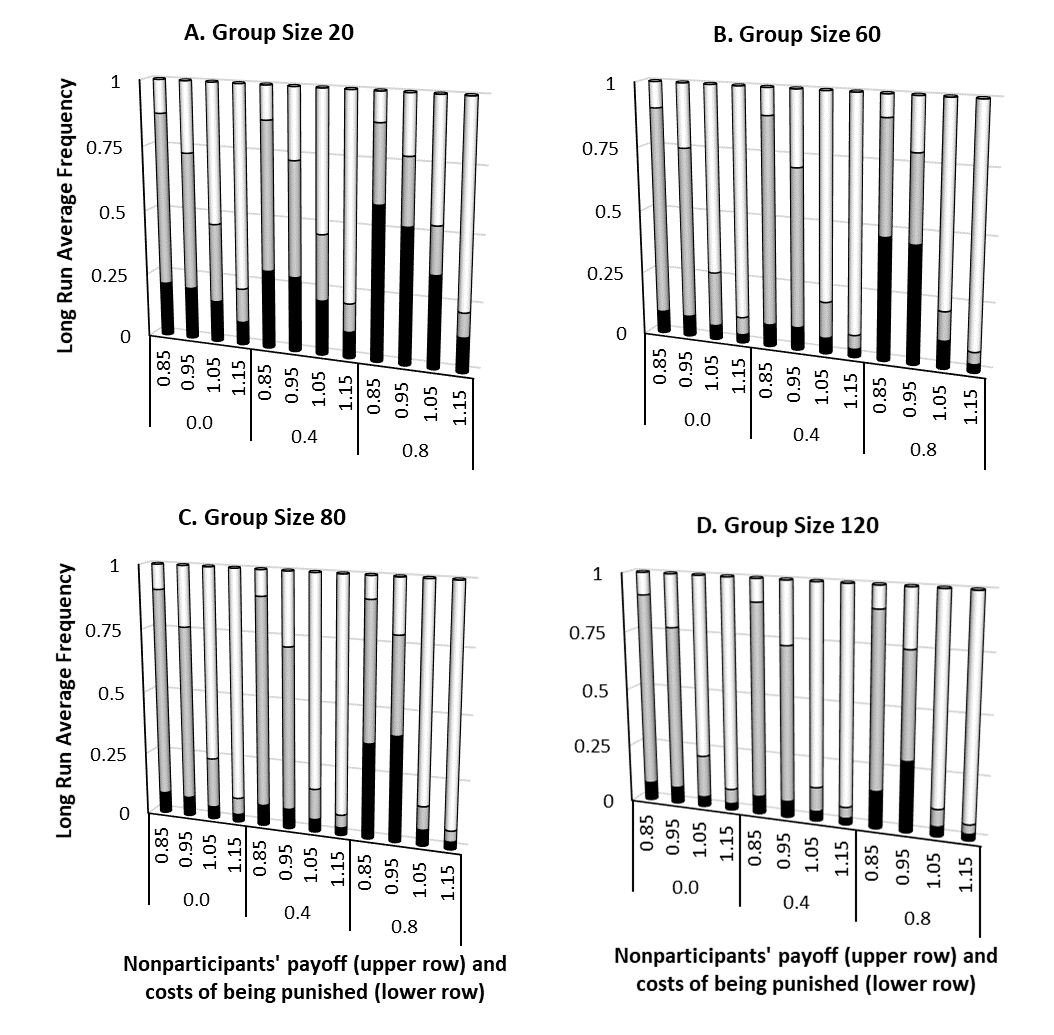


Fig S6. Variations in the long run average frequencies of strategies across nonparticipants’ payoffs (upper row on the X-axis) and the costs of being punished (lower row on the X-axis). Panels A to D depict the variations in the frequencies for different sizes of groups. The black cylinder indicates the frequency of cooperation (contributors and punishers), the gray the frequency of defection, and the white the frequency of nonparticipation.

Like the relatively low payoffs of nonparticipants (e.g., 0.85 and 0.95), the frequency of cooperation also remains very low at the high nonparticipants’ payoffs (e.g., 1.05 and 1.15) in the absence of punishment (square markers in Fig S5). However, such a marked decrease in cooperation is accompanied not by an increase in defectors but by the significant increase in nonparticipants (compare the last two gray and black bars at $p=0.0$ in Fig S6). This is because the nonparticipants’ payoffs such as 1.05 and 1.15 are high enough for both defectors and punishers to not participate in public goods provision. This means that both *defector-* and *punisher-decreasing* forces are so activated that variations between groups in the frequency of cooperation cannot be sufficient. Due to the lack of the substantial between-group differences, *group-selection* force contributing to the spread of cooperation cannot be activated.

In addition to the absence of punishment, we explored how decreasing the cost of being punished $(p=0.4)$ affects the long run average levels of cooperation. Fig S5 shows that the low cost of being punished generates the results which are almost the same as the results from the absence of punishment $\left( p=0.0 \right)$. This indicates that the cost of being punished, $p=0.4$, is not high enough to make a fundamental change in the dynamics of the three evolutionary forces towards increasing levels of cooperation.

References

1. Boyd R, Gintis H, Bowles S, Richerson PJ. The evolution of altruistic punishment. Proc Natl Acad Sci. 2003;100: 3531–3535. doi:10.1073/pnas.0630443100

2. Traulsen A, Hauert C, De Silva H, Nowak MA, Sigmund K. Exploration dynamics in evolutionary games. Proc Natl Acad Sci U S A. 2009;106: 709–712. doi:10.1073/pnas.0808450106
